# Integrating Systematic Reviews into Supportive Care Trial Design: The Rethinking Clinical Trials (REaCT) Program

**DOI:** 10.3390/curroncol29120750

**Published:** 2022-12-05

**Authors:** Bader Alshamsan, Brian Hutton, Michelle Liu, Lisa Vandermeer, Mark Clemons

**Affiliations:** 1 Department of Medicine, Division of Medical Oncology, The Ottawa Hospital, The University of Ottawa, Ottawa, ON K1H 8L6, Canada; 2 Department of Medicine, College of Medicine, Qassim University, Buraydah 52571, Saudi Arabia; 3 Clinical Epidemiology Program, The Ottawa Hospital Research Institute, University of Ottawa, Ottawa, ON K1H 8L6, Canada; 4 Cancer Therapeutics Program, Ottawa Hospital Research Institute, 501 Smyth Road, Ottawa, ON K1H 8L6, Canada

**Keywords:** breast cancer, systematic review, meta-analysis, network meta-analysis, pragmatic trial, clinical trial

## Abstract

Purpose: To review the successes and challenges of integrating systematic reviews (SRs) into the Rethinking Clinical Trials (REaCT) Program. Methods: All REaCT program SRs were evaluated and descriptive summaries presented. Results: Twenty-two SRs have been performed evaluating standard of care interventions for the management of: breast cancer (*n* = 15), all tumour sites (*n* = 4), breast and prostate cancers (*n* = 2), and prostate cancer (*n* = 1). The majority of SRs were related to supportive care (*n* = 14) and survivorship (*n* = 5) interventions and most (19/22, 86%) confirmed the existence of uncertainty relating to the clinical question addressed in the SR. Most SRs (15/22, 68%) provided specific recommendations for future studies and results were incorporated into peer-reviewed grant applications (*n* = 6) and clinical trial design (*n* = 12). In 12/22 of the SRs, the first author was a trainee. All SRs followed PRISMA guidelines. Conclusion: SRs are important for identifying and confirming clinical equipoise and designing trials. SRs provide an excellent opportunity for trainees to participate in research.

## 1. Introduction

The Rethinking Clinical Trials (REaCT) program is a novel Canadian-led clinical trials platform that focuses on comparing existing standard of care interventions for cancer management [1]. The program was established in 2014 to overcome many of the traditional challenges in clinical trial performance [2,3,4]. The REaCT process begins with end-user surveys to identify areas of clinical equipoise (i.e., topics involving varied clinical perspectives and a lack of consensus among the clinical community)and the identification research topics that patients, their families, and health care providers feel to be important [2,5,6,7].

**Figure 1:**
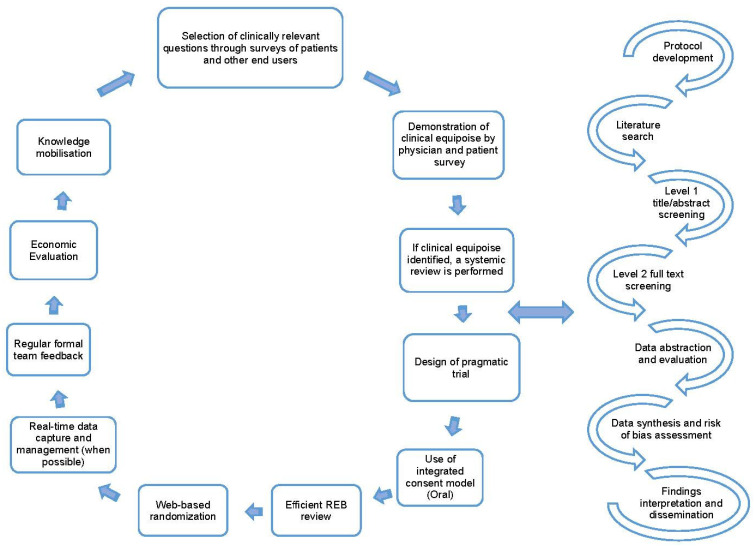
Key tenants for the REaCT Program; adapted with permission from [1,8] and the road map for systematic reviews.

Once clinical equipoise is identified, and the topic is deemed important to patient care, a literature search is performed to identify if an up-to-date systematic review on the topic is available. With these findings we then perform either an updated systematic review (when an older review is available) or a de novo review (when no prior reviews are identified) [1,8]. A systematic review is a comprehensive approach to reviewing the available evidence [9] and follow a well-established process that includes protocol development, search design and execution, level 1 and 2 screening of citations/full-text articles, data abstraction of study characteristics and outcomes, summary and synthesis of results, interpretation of findings, and dissemination of findings [10]. The key steps in the REaCT program process and the processes followed when performing systematic reviews are detailed in Figure 1.

Systematic reviews can lead to meta-analyses of outcome data to produce a single quantitative estimate from a synthesis of two or more studies [10]. The approach used to pool results from different studies depends on the similarity of the different study populations, the study methods and the clinical outcomes reported between the studies [11,12,13,14]. If, however, there are no overlapping clinical outcomes, or the presence of siginificant clinical heterogeneity in study populations evaluated in the different trials, descriptive methods for synthesis can also used [15]. Through these processes systematic reviews can provide a valuable summary of the available evidence for a research question of interest to patients, researchers, clinicians, and other stakeholders and can also be valuable in the process of trial design.

The current study evaluated the successes and challenges of integrating systematic reviews into the REaCT program. We also hope to demonstrate how systematic reviews should be a crucial step in the REaCT process, allowing us to confirm whether areas of patient and health care provider perceived clinical equipoise exist. In addition, we will show how performing a systematic review can assist in the design of potential clinical trials through the identification of previously unanswered questions and the choice of endpoints for such trials.

## 2. Materials and Methods

### 2.1. Search Strategy and Data Extraction

We reviewed all of the systematic reviews that have performed through the REaCT program from instigation until December 2021. As several co-authors (MC, BH, LV) have been involved in all the systematic reviews conducted by the REaCT program no formal database search to identify these reviews was required. The data was extracted by two authors (BA and MC) and reviewed by other team members (BH, ML, LV). The data extracted from each systematic review included: the primary research question, cancer care setting (e.g., adjuvant, metastatic, survivorship, palliative care), reporting strategy, approach to synthesis, and summary of findings. In addition, data was collected evaluating: the number of abstracts screened (phase I), articles (phase II) and the included studies, number of authors (staff and trainees), and training level of the first author (resident, fellow, staff) for each of the systematic reviews.

### 2.2. Review Outcomes

The authors were interested in whether or not the individual systematic reviews identified, confirmed or answered the clinical questions identified by stakeholders (patients, their families and healthcare provider surveys). Furthermore, the outcome of each systematic review was also sought, including recommendations to apply for peer-reviewed grant funding to answer the particular clinical question as well as if systematic review affected future and clinical trial design, and/or the decision to try and perform a definitive trial. Descriptive summaries were used to present the study findings.

## 3. Results

### 3.1. Reviews Characteristics

**Figure 2:**
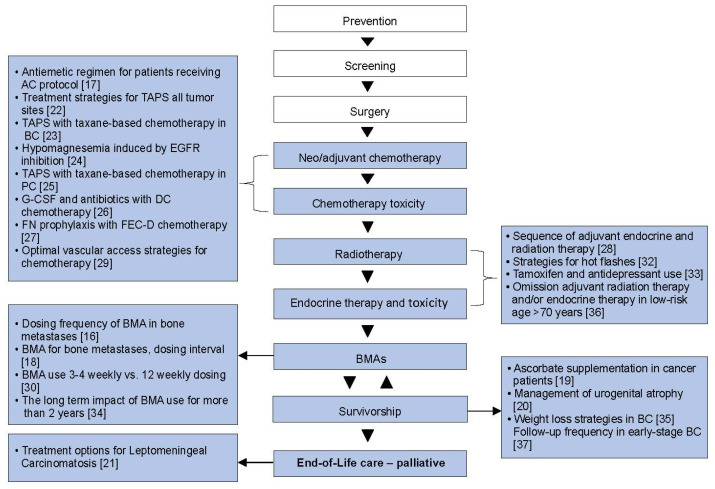
The cancer journey: where the systemic reviews fit. BC, breast cancer; BMAs, bone-modifying agents; DC, docetaxel–cyclophosphamide; EGFR, epidermal growth factor receptor; FEC-D, 5-fluorouracil, epirubicin; PC, prostate cancer; TAPS = taxane-associated pain syndrome [16,17,18,19,20,21,22,23,24,25,26,27,28,29,30,32,33,34,35,36,37].

Out of 28 areas of clinical equipoise identified from surveys of patients and healthcare providers [7], 22 systemic reviews have been performed and published [16,17,18,19,20,21,22,23,24,25,26,27,28,29,30,31,32,33,34,35,36,37]. Of these, 18 were new reviews, while 4 were updated reviews of previously published reviews. The reviews were related to areas of clinical equipoise in: breast cancer (*n* = 15), all tumour sites (*n* = 4), breast and prostate cancers (*n* = 2), and prostate cancer (*n* = 1) management. These reviews covered topics regarding; adjuvant therapy [28,36], adjuvant supportive care [17,21,26,27,29], metastatic [21], palliative supportive care [16,18,25,30,34], both adjuvant and palliative supportive care [19,22,23,24,31], and survivorship [20,32,33,35,37]. The details of each review are shown in Table 1, and where each question lay in the cancer journey is shown in Figure 2.

The median number of abstracts assessed for phase I screening was 1447 (range, 113–3860), while a median of 65 (range, 7–640) full-text articles were reviewed for phase II screening. The median number of included articles was 11 (range, 4–173). All reviews followed Preferred Reporting Items for Systematic Reviews and Meta-Analyses (PRISMA) guidelines. However, one review did not specify the reporting guidelines, but the methodology was consistent with PRISMA guidelines [24]. The median number of authors in each publication was 12 (range, 5–18), and of these, 5 (range, 1–9) were trainees (i.e., a resident or fellow). The first author was a trainee in 54% (12/22) of reviews.

### 3.2. Impact of Systemic Reviews

Most of the systematic reviews (19/22, 86%) confirmed the presence of clinical equipoise, and only three provided strong recommendations [25,27,30]. LeVasseur et al. conducted a systematic review with network meta-analyses that included 98 randomised controlled trials (RCTs) and concluded that dietary and combination interventions of diet and exercise significantly improved anthropometric measures compared to standard care [35]. Surujballi et al. performed a systematic review that included six RCTs and one prospective cohort study, and concluded that reduced frequency of follow-up of early stage breast cancer has no adverse effects on breast cancer-related outcomes [37]. Bradbury et al. conducted a systematic review that included 15 studies (2 RCTs, 10 retrospective cohorts, and 3 case–control) that concluded that concurrent use of tamoxifen and antidepressants is most likely safe for breast cancer-related outcomes [33].

**Figure 3:**
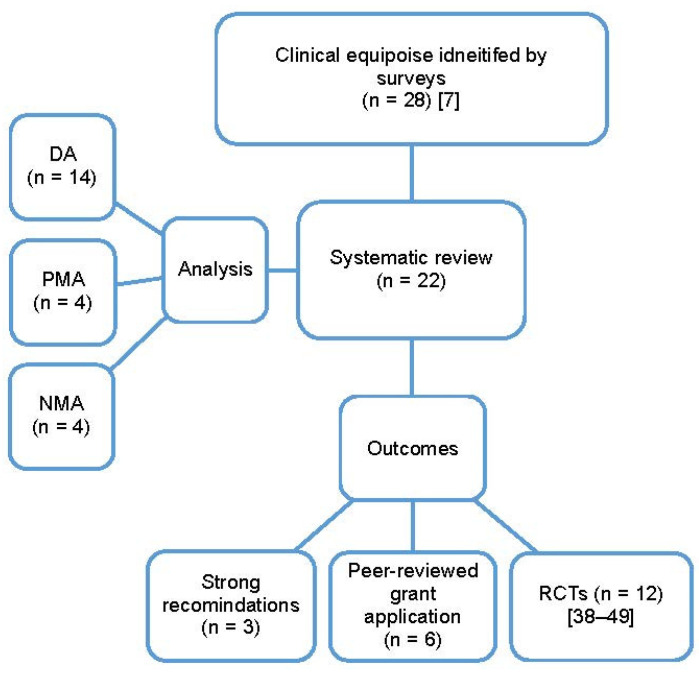
The type of analysis and outcomes of the systematic reviews performed and published based on clinical equipoise identified by surveys; DA, descriptive analysis; NMA, network meta-analysis; PMA, pairwise meta-analysis; RCTs, randomized clinical trials [7,38,40,41,42,43,44,45,46,47,48,49,50].

The cited factors that led to the inability to provide strong recommendations from systematic reviews included: heterogeneity in the reporting of outcomes between the included studies (16/22, 72%), differences in study types and populations (14/22, 63%), as well as the lack of reporting or variation in the reporting of variables potentially associated with different outcomes (16/22, 72%). Other limitations included the paucity of relevant published studies (10/22, 45%), and the lack of RCT data (7/22, 31%). Most of the systematic reviews performed cited that multiple limiting factors to the interpretation of data were present. Most studies (*n* = 14) used a descriptive approach to synthesis due to clinical, methodologic and/or statistical heterogeneity amongst the included studies. There was sufficient data for 4 reviews with pairwise meta-analysis [17,20,32,35] and 4 for network meta-analyses [18,30,33,36]. For all the systematic reviews performed, the authors recommended that further studies were needed, even for those that provided a firm conclusion. Reasons for further studies included: specifically designed RCT to measure the magnitude of benefit with different measures [35], to strengthen the current evidence [33], and to assess the impact of health economic aspects [37]. In 15 (68%) reviews, the authors suggested specific recommendations for future clinical trials; the findings of these reviews were incorporated into 6 peer-reviewed grant applications and the design of 12 clinical trials (6 RCTs were published [42,43,44,45,48,50], and 6 are ongoing [38,40,41,46,47,49]), (Figure 3).

## 4. Discussion

The Rethinking Clinical Trials (REaCT) Program was created to overcome many of the barriers in traditional clinical trial design and performance in oncology. The REaCT program is the largest pragmatic cancer clinical trials program in Canada, with more than 4000 patients participating in clinical trials at 16 Canadian centres [51]. As part of the REaCT process, systematic reviews are a key step to address whether areas of patient- and health care provider-perceived clinical equipoise exist. Systematic reviews can also help in the design of potential future clinical trials through the identification of previously unanswered questions as well as challenges faced in previous studies and the choice of endpoints for such trials. As a result most topics showing clinical equipoise that were identified from surveys of patients and health0care providers [7] resulted in 18 new systematic reviews and 4 updates of previously published systematic reviews. So far, these reviews have led to 6 peer-reviewed grant applications and 12 RCTs [38,40,41,42,43,44,45,46,47,48,49,50]. The involvement of trainees in these reviews was important and in almost half of these reviews, trainees took a senior role in the particular review.

It is important to note that in the majority of systemic reviews, meta-analysis was not feasible, and in most reviews (14), authors used a descriptive approach to synthesize the available data. These numbers reflect the limitations of using the published literature to answer many specific clinical questions. The major limitations of the evidence base identified for synthesis included heterogeneity in; study types, populations and reporting outcomes; as well as the lack of unity in the definitions of study outcomes and the lack of reporting on variables and risk factors that can have important implications for a particular study outcome. Further limitations included the limited number of published studies, and the lack of relevant randomized controlled trials. As a result of all of these variables most reviews are unable to provide firm recommendations.

Despite these limitations all the perfomed systematic, reviews were able to highlighted areas of limitations and knowledge gaps in current studies. Not surprisingly the majority of reviews made specific recommendations that future clinical trials if clinical equipoise was to be resolved. For example, Hutton et al. provided a comprehensive review on identifying the optimal antiemetic regimen for patients receiving anthracycline and cyclophosphamide-based chemotherapy. Their systematic review identified 47 different antiemetic regimens and 15 different chemotherapy-induced nausea and vomiting endpoints that were used in these trials. They recommended that future trialists unify emesis outcome definitions and balance patients for risk factors reated to underlying emesis risk [17]. For another example, Fernandes et al. recommended that future studies use validated scores to measure taxane acute pain syndrome to evaluate treatment response [22]. In a review comparing bone modifying agents frequency (q3–4 weeks vs. 12 weeks) in breast cancer patients with bone metastasis, Awan et al. recommended future studies to stratify patients based on bone vs. visceral disease, disease burden, bone metastasis site, and bone turnover markers to allow decreased heterogenicity between trials [30].

## 5. Conclusions

Systematic reviews provide an important tool in the performance of clinic research. The current manuscript shows how incorporation of systemic review into The REaCT program continues to confirm that in many areas of clinical care identified by patients, their families and healthcare providers that insufficient evidence for optimal practice exist and therefore clinical equipoise is present. Systemic reviews also provide an important tool for designing future potential clinical trials by exploring the challenges and limitations reported in previously reported studies. Systematic review also provide unique opportunities for trainees to be fully incorporated into the research process.

## Figures and Tables

**Table 1:** Summary of the systematic review (*n* = 22).

Phase of Cancer Journey	References	Cancer Type	Topic/Question	Type of Analysis	Synopsis of Review Findings	The Systemic Review Led to
Adjuvant	[28]	BC	Sequence of adjuvant ET and RT in early-stage BC	DA	Concurrent treatment appears safe. Further studies are needed to evaluate treatment-related toxicities.	REaCT-RETT [38]
	[36]	BC	Omission of adjuvant RT and/or ET in older patients treated with BCS for low-risk BC	MA	RT can be omitted in older patients with lower-risk diseases.	REaCT-70 [39]
Adjuvant Supportive care	[17]	BC	Optimal antiemetic regimen for patients receiving anthracycline and cyclophosphamide-based chemotherapy for BC	NMA	High variability in the outcomes reported by individual RCTs. Identifying an optimal antiemetic regimen was not possilbe.	REaCT-ILIAD [40]
	[26]	BC	G-CSF and antibiotics use for primary FN prophylaxis in patients receiving DC chemotherapy for BC	DA	Insufficient data to make a recommendation of one strategy over another (G-CSF vs. antibiotics)	REaCT-TC and TC2 [41]
	[27]	BC	Primary FN Prophylaxis for Patients who receive FEC-D chemotherapy for BC	DA	Identification of the optimal choice and timing of primary FN prophylaxis was not possible.	REaCT-G and G2 [42]
	[29]	BC	Optimal vascular access strategies for patients receiving chemotherapy for early-stage BC	DA	The published evidence identifying the optimal type of venous access is weak.	REaCT-VA Her2 Negative [43]
Metastatic	[21]	BC	Treatment options for leptomeningeal carcinomatosis in BC patients	DA	Limited high-quality evidence exists regarding the optimal treatment of LC-BC	Recommend RCT
Palliative Supportive care	[16]	BC	Dosing frequency of BMA for patients with bone metastases from breast cancer	DA	The benefits of standard treatment compared to de-escalated therapy for commonly used BMA requires further study.	Recommend RCT
	[18]	BC	4-weekly of BMA vs. de-escalated (Q12-weekly) dosing in BC patients with bone metastases.	MA	No difference in SREs or pain with de-escalated therapy.	REaCT-BTA [44,45]
	[25]	PC	TAPS in PC patients who have received taxane-based chemotherapy	DA	Quantified the incidence of TAPS and contributed to explaining the potential risks of developing TAPS in PC.	Recommend RCT
	[30]	BC	Efficacy and harms of standard 3–4-weekly versus 12-weekly dosing of BMAs in breast cancer patients with bone metastases	MA	The literature supports de-escalation of zoledronate from the onset for patients with bone metastases from breast cancer.	REaCT-BTA [44,45] REaCT-ZOL [46]
	[34]	BC&PC	The long-term impact of BMA use for >2 years in BC or CRPC for the treatment of bone metastases	DA	No high-quality evidence to support the use of BMA for more than two years.	REaCT-HOLD BMA [47]
Both Adjuvant/Palliative Supportive care	[19]	All cancer types	Administration of oral or IV ascorbate in cancer patients	DA	No evidence to suggest that ascorbate in cancer patients either enhances the antitumor effects of chemotherapy or reduces its toxicity	Recommend RCT
	[22]	All cancer types	Treatment strategies for TAPS across all tumour sites	DA	TAPS remains poorly researched. Fw studies evaluate its optimal management.	Recommend RCT
	[23]	BC	TAPS in BC patients who have received taxane-based chemotherapy	DA	The incidence of TAPS varies between taxanes, regimens, and disease settings.	REaCT-TAPS [48]
	[24]	All cancer types	Evaluating interventions on hypomagnesemia induced by EGFR inhibition	DA	There is an absence of high-quality evidence for the management of EGFRI-induced hypomagnesemia.	REaCT-Mg [49]
	[31]	All cancer types	Benefits and harms of cannabis-based medicines	DA	It is possible that the harms of cannabis-based medicines may outweigh the benefits	Recommend RCT
Survivorship	[20]	BC	Management of urogenital atrophy in breast cancer patients	NMA	Treatment of urogenital atrophy remains a challenging issue.	Recommend RCT
	[32]	BC&PC	Management of hot flashes in BC or PC patients	NMA	Many interventions may offer improvements for HFs versus no treatment, but no optiml therapy idenified.	Recommend RCT
	[33]	BC	Concurrent tamoxifen and antidepressant use	MA	The totality of evidence suggests that concurrent antidepressant and tamoxifen is likely safe.	Conclusion
	[35]	BC	Weight loss strategies in patients with early-BC	NMA	Diet and exercise alone or in combination are effective lifestyle interventions.	Conclusion
	[37]	BC	Frequency of follow-up visits for patients with early-stage BC	DA	Reduced frequency of follow-up has no adverse effects on BC outcomes.	Conclusion

BC, breast cancer; BMAs, bone-modifying agents; DA, descriptive analysis; DC, docetaxel–cyclophosphamide; EGFR, epidermal growth factor receptor; ET, endocrine therapy; FEC-D, 5-fluorouracil, epirubicin; FN, febrile neutropenia; PC, prostate cancer; TAPS, taxane-associated pain syndrome; NMA, network meta-analysis; PMA, pairwise meta-analysis; RCTs, randomized clinical trials; SREs, skeletal-related events.

## Data Availability

All data was extracted from published manuscripts.
